# The Universal Food

**Published:** 1879-08

**Authors:** L Larrken


### *THE UNIVERSAL FOOD.*

By L Larrken, M.D.

The chemist of the Health Food Company has occupied many months in perfecting this food. His idea has been to prepare a food which should possess in a superior degree these essential qualities, viz., an agreeable odor and a palatable flavor; entire freedom from inert and insoluble particles; richness in the nitrogenous and phosphatic albumenoids; ready assimilability, and a completion of the cooking processes. The result is a strong blood-making and waste-repairing food, adapted to the infant, the adult, and the aged, ready for instant use, and capable of being easily and quickly digested, with the smallest possible demand upon the fluids and machinery involved. It is not claimed to be a partly digested food—for that is an impossibility; but it is fully prepared for digestion, and awaits only the appropriate conditions—the admixture of a suitable fluid vehicle and exposure to the juices of the stomach, to insure it. We have proved by several careful experiments that if mixed with three or more volumes of milk or water in a glass tube, at a temperature of 98° Fahr., it is converted into blood-substance in from forty to fifty minutes by the addition of one volume of fresh gastric juice; the resulting fluid being closely allied, chemically, to pure arterial blood.

There are several considerations connected with this scientific food which may be profitably discussed. In the first place, the Universal Food is packed for shipment in the form of a dry powder. Dryness is an absolute necessity when concentration is sought. We can dilute, attenuate, by adding fluid, but we cannot thus concentrate. This food is made dry, therefore, for reasons which all will appreciate; first, for convenience in handling and transporting: secondly, to secure the greatest possible concentration; and, thirdly, to render it imperishable for all time, under all ordinary conditions of exposure. Had it been offered as a fluid, it would have been necessarily weak in food-constituents, and would have demanded alcohol or other deleterious antiseptic to preserve it from destructive fermentation. An amount of nutriment equal to that which is contained in one package of this Universal Food, would occupy as good cow's milk, 40 quarts, and would equal in blood-making power, 60 eggs, 10 lbs. beef or mutton, 30 lbs. wheat, and 100 lbs of soda-crackers. In either of these forms it would have been bulky, because associated with a vast preponderance of either inert or injurious matter, or matter easily obtainable everywhere when desired to be added. The fluid part of all real food is, and ever must be, water; and water is, happily, forever accessible. It is therefore carefully excluded from this food.

We say this food is perfectly cooked. Now, the object of cooking is not simply to impart a pleasant flavor; it is equally to convert indigestible substances into digestible ones. The inadequate or imperfect performance of that process of vital import—the cooking process—not unfrequently constitutes the sole difference between injurious food and admirable food. Aware of this truth, and that facilities for perfect cooking are not always at hand, they thoroughly cook their Universal Food, and thereby render it impossible for the careless cook to impair either its flavor, its digestibility, or its nutritive value. That the cook has the power to ruin all, is sufficiently attested by the fact that the exposure of any known food-substance to a temperature of carbonizing power, burns up the nutritive albumen, volatilizes the phosphatic salts, and converts the fatty and other carbons into empyreuma—a deadly enemy of digestion. Wisely, therefore, this concentrated Universal Food presents itself in a cooked form, precisely as does the more diluted food—milk. The cooking process is carried on for thirty hours, at a temperature of 160°, and the result is a perfect food for all ages and conditions of humanity. It is the true cure for many unnatural digestive states, and is capable of supporting life at its best with no assistance from other foods. In diabetes alone should its use be avoided.

